# Dataset on negative symptoms factors in patients with schizophrenia

**DOI:** 10.1016/j.dib.2022.107790

**Published:** 2022-01-05

**Authors:** T.V. Lezheiko, N.Yu. Kolesina, V.E. Golimbet

**Affiliations:** Mental Health Research Center, Kashirskoe sh.34, Moscow 115522, Russian Federation

**Keywords:** Schizophrenia, Season of birth, PANSS, Negative symptoms factors

## Abstract

Schizophrenia is a severe mental disorder characterized by positive and negative symptoms. The negative symptoms are highly relevant to the disease course and outcome. Because negative symptoms show considerable heterogeneity, there is substantial interest in elucidating the negative symptom domains that are characteristic of patient subgroups. It has been proposed that patients with schizophrenia should be classified into deficit and non-deficit groups based on the severity of their negative symptoms. Another method suggested the assessment of the factor structure of negative symptoms to understand its mechanisms. Factor analysis of the different negative symptom rating scales reveals two distinct negative symptom subdomains: diminished expression (DE) and avolition/apathy (AA). These characteristics suggest different pathophysiological mechanisms for the development of AA and DE. We present a large dataset of negative symptom factors calculated for 3006 patients with schizophrenia in the Russian population. Sex, age, age at disease onset and data of birth, including season of birth (SOB), family history of schizophrenia are presented. Negative symptoms were assessed with the Positive and Negative Syndromes Scale (PANSS). We calculated negative symptoms factors as suggested by Liemburg et al. (2013). The data will be useful in assessing the impact of such factors as sex, season of birth (SOB) and family history on the scores of negative symptoms subdomains; such data can help us to better understand the heterogeneity of the negative symptoms of schizophrenia.

## Specifications Table

**Table:** 

Subject	Psychiatry
Specific subject area	Schizophrenia, risk factors.
Type of data	Tables, Excel file: demographics, clinical characteristics, information on family history of schizophrenia.
How the data were acquired	Selection of patients was based on the following criteria: diagnosis of schizophrenia established by a structured psychiatric examination (The Mini International Neuropsychiatric Interview), informed consent to participate in the study. Assessment of symptoms of schizophrenia was performed with the Positive and Negative Syndromes Scale (PANSS). Information about demographics, clinical characteristics and family history of schizophrenia was obtained from medical histories and structured interviews.
Data format	Raw, analyzed.
Parameters for data collection	Adults (+18 years old) living in Moscow and Moscow region. PANSS to measure schizophrenia symptoms. Independent factors: demographic and clinical characteristics, family history of schizophrenia.
Description of data collection	Schizophrenia symptoms were assessed with PANSS. For diminished expression (DE) and avolition/apathy (AA) subdomains calculation PANSS items were used as suggested by Liemburg et al (2013).
Data source location	Mental Health Research Center, Moscow, Russia.
Data accessibility	The data is available within this article.

## Value of the Data

• The dataset provides scores on subdomains of negative symptoms of schizophrenia derived from PANSS and factors relevant for the study of negative symptoms structure in schizophrenia.
• The data can be used in meta-analyses of the clinical characteristics of schizophrenia and will be valuable for epidemiological studies of the disease.
• The data will be useful in assessing the impact of such factors as sex, season of birth (SOB) and family history on the scores of negative symptoms subdomains; such data can help us to better understand the heterogeneity of the negative symptoms of schizophrenia.
• The data will be of interest to researchers studying the neurobiological differences underlying negative symptoms domains.

## 1 Data Description

Schizophrenia is a severe mental disorder characterized by positive and negative symptoms. The negative symptoms are highly relevant to the disease course and outcome [1]. Because negative symptoms show a considerable heterogeneity, there is a prominent interest in elucidation of domains characteristic of subgroups of patients. Because negative symptoms show considerable heterogeneity, there is substantial interest in elucidating the negative symptom domains that are characteristic of patient subgroups. It has been proposed that patients with schizophrenia should be classified into deficit and non-deficit groups based on the severity of their negative symptoms. Deficit patients and nondeficit patients differ in 5 disease dimensions: signs and symptoms, the course of illness, treatment response, biological correlates and risk/etiological factors. The risk factors for DS are male gender, a family history of schizophrenia and being born in the summer [3]. Another method suggested for studying the mechanisms of negative symptoms is to assess the factor structure of negative symptom scales [4], [5]. Factor analysis of the various rating scales of negative symptoms reveals two distinct negative symptoms subdomains: diminished expression (DE) and avolition/apathy (AA) [5], [6], [7]. Some studies show differential effects of these subdomains on clinical features, disease progression and functional outcomes of schizophrenia (for review see [8]). Symptoms in the AA subdomain are associated with poorer functioning, a longer duration of untreated psychosis and non-adherence to treatment. Symptoms in the DE subdomain are more persistent over time, more prevalent in the early stages of the disease, and associated with a longer duration of the prodromal phase of the disease than those in the AA subdomain [8]. These characteristics suggest different pathophysiological mechanisms for the development of AA and DE [2], [4].

Here we present a large dataset of negative symptom factors calculated for patients with schizophrenia in the Russian population. Negative symptoms were assessed with PANSS. We calculated negative symptoms factors as suggested in [6]. PANSS-derived AA factor consisted of Emotional withdrawal (PANSS item N2), Apathetic social withdrawal (N4), Active social avoidance (G16). DE factor included Blunted affect (N1), Poor rapport (N3), Lack of spontaneity (N6), Mannerism and posturing (G5), Motor retardation (G7), Disturbance of volition (G13). The dataset contains data for the period 2007 to 2020. Sex, age, age at disease onset and data of birth, including season of birth (SOB), are presented. Scores on the DE and AA subdomains are available for all patients, SOB for 2492 patients and presence or absence of a family history of schizophrenia for 1986 patients. Minimal and maximal values as well as median, mean and standard deviation (SD) for each factor are presented in Table 1. Table 2 describes demographic (sex, age, SOB), clinical (age at disease onset) and family history characteristics of the DE and AA subdomains.

**Table 1:** Statistical measures of negative symptoms factors in the studied sample of patients with schizophrenia.

Statistical measures	AA factor	DE factor
Minimum	2.0	6.0
Median	9.0	17.0
Maximum	21.0	41.0
Mean	9.5	17.2
SD	3.9	5.8

**Table 2:** Characteristics of negative symptoms factors in the studied sample of patients with schizophrenia.

Sample characteristics	AA, mean (SD)	DE, mean (SD)
Men (n=898)	9.6 (3.5)	17.3 (5.4)
Women (n=2108)	9.5 (4.1)	17.2 (6.0)
Family history of schizophrenia Yes (n=674) No (n=1312)	9.6 (4.0) 9.5 (3.8)	17.7 (6.0) 17.5 (5.7)
Winter birth Yes (n=646) No (n=1848)	9.8 (4.1) 9.2 (3.8)	17.3 (5.9) 16.8 (5.6)
Summer birth Yes (n=647) No (n=1848)	9.3 (3.8) 9.4 (4.0)	17.0 (5.6) 16.9 (5.6)
Autumn birth Yes (n=647) No (n=1848)	9.2 (3.8) 9.4 (3.9)	16.7 (5.2) 17.0 (5.8)
Spring birth Yes (n=647) No (n=1848)	9.2 (3.9) 9.4 (3.9)	16.7 (5.8) 17.0 (5.6)

## 2 Experimental Design, Materials and Methods

### 2.1 Sample collection

The schizophrenia sample was selected from patients admitted to psychiatric units of the Mental Health Research Center. It included 3006 people (898 men, 2108 women) with a mean age of 38.4 (SD 13.6) years and mean age at disease onset of 26.4 (SD 11.1) years. All participants provided written informed consent for participation in the study.

### 2.2 Phenotyping

The diagnosis of schizophrenia was made according to criteria of the International Classification of Diseases 10th revision (ICD-10) and confirmed by a structured psychiatric examination (the Mini International Neuropsychiatric Interview) [9]. Clinical symptoms were measured using PANSS, a widely used instrument proven to be valid and suitable for the evaluation of the positive, negative and general psychopathology of schizophrenia. PANSS includes three subscales, totaling 30 items: 7 relating to positive, 7 to negative and 16 to general psychopathological symptoms. Each symptom is rated on a scale of 1 to 7 (1– absent, 2– questionable, 3 – mild, 4 – moderate, 5 – severe, 6 – markedly severe, 7 – extremely severe). PANSS was administered by a professionally trained and experienced psychiatrist. The patients were from several clinical departments, and there were different interviewers in each. The PANSS interviews were conducted one week before the patient's discharge from the hospital.

Supplementary material is presented as a two-sheet Excel spreadsheet file. Sheet 1 contains columns recording the clinical department designation, patient identification number (ID), family history (yes, no), sex (female, male), date of birth, summer birth (yes, no), winter birth (yes, no), autumn birth (yes, no), spring birth (yes, no), age in years at admission, age in years at onset, scores on the PANSS items, AA scores and DE scores. Sheet 2 contains descriptions of the PANSS items.

## Ethics Statement

The study was conducted in accordance with the Declaration of Helsinki, and the study protocol was approved by the local Ethics Committee (No 98 of 11.09.2007).

All participants provided the written informed consent for participation in the study.

## CRediT authorship contribution statement

**T.V. Lezheiko:** Formal analysis, Investigation, Data curation, Writing – original draft. **N.Yu. Kolesina:** Investigation, Data curation. **V.E. Golimbet:** Conceptualization, Writing – review & editing, Supervision.

## Declaration of Competing Interest

Authors declare that there is no competing interest.

## Appendix Supplementary materials

Image, application 1
